# A vector dataset of the high-speed road network in Poland integrated with commissioning year information (1936–2023)

**DOI:** 10.1016/j.dib.2026.113235

**Published:** 2026-09-03

**Authors:** Katarzyna Krasnodębska, Przemysław Śleszyński, Johannes H. Uhl, Łukasz Kubiak

**Affiliations:** a Institute of Geography and Spatial Organization, Polish Academy of Sciences, Warsaw, Poland; b European Commission, Joint Research Centre (JRC), Ispra, Italy

**Keywords:** Motorway network evolution, Expressway, Historical GIS, Long-term landscape change, Data conflation, Transport, Poland, European Union

## Abstract

The High-Speed Road Network Vector Data with Construction Year Attribution for Poland (HiRoN-PL) provide spatially and temporally explicit data documenting the development of the high-speed road network in Poland between 1936 and 2023. The dataset is distributed in GeoPackage format containing vector line features representing the time-stamped high-speed road network in Poland. HiRoN-PL road geometries are derived from OpenStreetMap (OSM) ‘*way’* objects corresponding to high-speed road classes in Poland, namely motorways and expressways (dual carriageways). The selected OSM road sections were aligned with authoritative data provided by the General Directorate for National Roads and Motorways (GDDKiA), the central authority responsible for national roads in Poland. First, the authoritative GDDKiA road geometries were attributed with commissioning dates based on expert interpretation of information from GDDKiA records, public registers, and verified crowdsourced data sources. Second, the geometry of OSM road sections was adjusted to reflect distinct construction phases as defined by the authoritative data. The resulting OSM-derived road network was then assigned with temporal attributes, including the year, month, and day of commissioning, as well as the road segment name, class, and the names of the road junctions that designate the starting and ending nodes of each segment. The dataset includes additional attributes, such as the length of each section and identifiers linking to the source OSM and authoritative data. HiRoN-PL allows for spatial-historical analyses of transport infrastructure development at a national level, enabling multi-temporal accessibility modelling and assessment of transportation efficiency over time. The dataset can be used to support analyses related to the monitoring of land use changes, the impact of transport and planning policies, or the effects and impacts of European Union investment policies. Data (version 1.1) are publicly available at https://doi.org/10.5281/zenodo.19570041.

Specifications Table

**Table:** 

Subject	Earth & Environmental Sciences
Specific subject area	Geography and Spatial Science
Type of data	HiRoN-PL (High-Speed Road Network Vector Data with Construction Year Attribution for Poland) dataset (current version v1.1) includes a GeoPackage file (hiron-pl_2180_v1_1.gpkg) containing vector line features representing attributed high-speed road network in Poland.
Data collection	Road geometries were generated by selecting from Open Street Map (OSM) ‘*way*’ objects features corresponding to classes of high-speed roads. The OSM road geometries were aligned to distinct commissioning phases. The positions of the start and end points of road sections were adjusted to match the road geometries obtained from the General Directorate for National Roads and Motorways (GDDKiA), the central authority of national roads in Poland. The adjusted road sections were enriched with commissioning dates derived from the authoritative and crowdsourced sources; OSM and GDDKiA identifiers, road segment name, length, class and the names of the start and end junctions of individual segments.
Data source location	•The OSM road geometries OSM road geometries for Poland were extracted from the OSM data archive, downloaded from the Geofabrik server on 19 January 2026 (https://download.geofabrik.de/europe/poland.html). •The authoritative geometry and attributes of high-speed road segments Authoritative data were derived from the General Directorate for National Roads and Motorways (GDDKiA), the central authority responsible for national roads in Poland, which manages and monitors high-speed road network construction. A register of the road network status is available via website *Mapa Stanu Budowy Dróg* (‘*Road Construction Status Map’*, https://www.gov.pl/web/gddkia/msbd). Spatial extent of the collected data: [14.2505296894545, 49.425001682562, 23.8638251777254, 54.4856843837452]
Data accessibility	Repository name: Śleszyński, P., & Krasnodębska, K. (2026). HiRoN-PL: High-Speed Road Network Vector Data with Construction Year Attribution for Poland (1936-2023) (v1.1) [Data set]. Zenodo. Version-specific identifier: 10.5281/zenodo.19570041 Direct URL to data: https://zenodo.org/records/19570041 The authoritative road network data used in the production of the HiRoN-PL v1.1 dataset were obtained from GDDKiA following a request by the authors. These data are not publicly available online, however, they can be obtained directly from GDDKiA upon request. The results of the expert interpretation of the commissioning phases based on these authoritative road network data, used as an intermediate step in the development of HiRoN-PL v1.1, are available from the authors upon request.
	All data processing was performed using Python 3.10 with open-source packages, including GeoPandas, Shapely, NumPy, and NetworkX. OpenStreetMap data were extracted using the Osmium tool and converted to GeoPackage format using GDAL/OGR. All spatial operations were conducted in the National Geodetic Coordinate System 1992, ETRS89 / Poland CS92 (EPSG code: 2180), a projected coordinate reference system based on the ETRF2000-PL (ETRS89) datum and the GRS 1980 ellipsoid, using the Transverse Mercator projection. Underlying code is available at: https://github.com/katarzynagoch/hironpl
Related research article	None.

## 1 Value of the Data

• The HiRoN-PL v1.1 dataset [1] reflects nearly a century of road infrastructure development in Poland. Following the construction of the first motorways before the Second World War and the subsequent period of stagnation, there was a significant expansion of the high-speed^1^ road network after the country joined the European Union in 2004, and this process accelerated during the preparation for the 2012 UEFA European Football Championship, organized jointly by Poland and Ukraine. The HiRoN-PL dataset is therefore a valuable source of information for analysing the long-term development of road infrastructure at national and regional level.
• The HiRoN-PL v1.1 dataset provides a basis for comprehensive analyses of the effects of infrastructure policy of the European Union (EU). Poland is one of the largest beneficiaries of the EU cohesion policy in terms of structural funds allocated to the transport sector [2,3]. Given the huge investment needs, extensive development of the road network were carried out under both the 2007–2013 and 2014–2020 EU funding periods. The availability of the temporally attributed high-speed road network data allows for a comprehensive assessment of the impact of these investments on changes in transport accessibility, its spatial distribution, and its impact on territorial cohesion, whilst also enabling the precise identification of new investment priorities [4].
• Due to its wide spatial coverage, detailed vector geometry, and consistent temporal attribution, HiRoN-PL v1.1 provides a valuable reference source for remote-sensing based historical road network extraction [5]. HiRoN-PL data, covering the dynamic development of road infrastructure over the past two decades, coincides with a period of increased availability of satellite observations. Therefore, it is a particularly suitable testbed for applications covering a region characterized by rapid infrastructure changes where reference data are typically uncertain or scarce.

## 2 Background

High-speed road networks are a key element of transportation infrastructure, enabling the efficient movement of people and goods between regions and countries. Data on the evolution of the high-speed roads are critical to understand the relationship between road network structure and economic features [6], to assess the effects of national and international transport policies [4], or to measure the impact of transport infrastructure on the spatial patterns of accessibility indicators [7,8].

While the evolution of local, residential road networks can be inferred from the evolution of nearby settlements [[9], [10], [11], [12]], the digital data measuring the temporal evolution of high-speed (or ‘*motorway’*, ‘*highway’*) road networks from national mapping agencies are rare (e.g. the TIGER/Line dataset by US Census Bureau [13]). Manual digitization of historical road maps allows for a controlled assignment of road construction epochs, but it is labour-intensive and its spatial accuracy depends on the resolution of historical sources [14]. In current approaches, abundant contemporary road network data can be automatically combined with information from historical maps to estimate their age [15]. We combine these two approaches in HiRoN-PL v1.1 and, through expert interpretation, enrich open spatial data on road geometry from OSM with temporal attributes derived from public registers of motorway and expressway construction in Poland.

## 3 Data Description

**Fig. 1:**
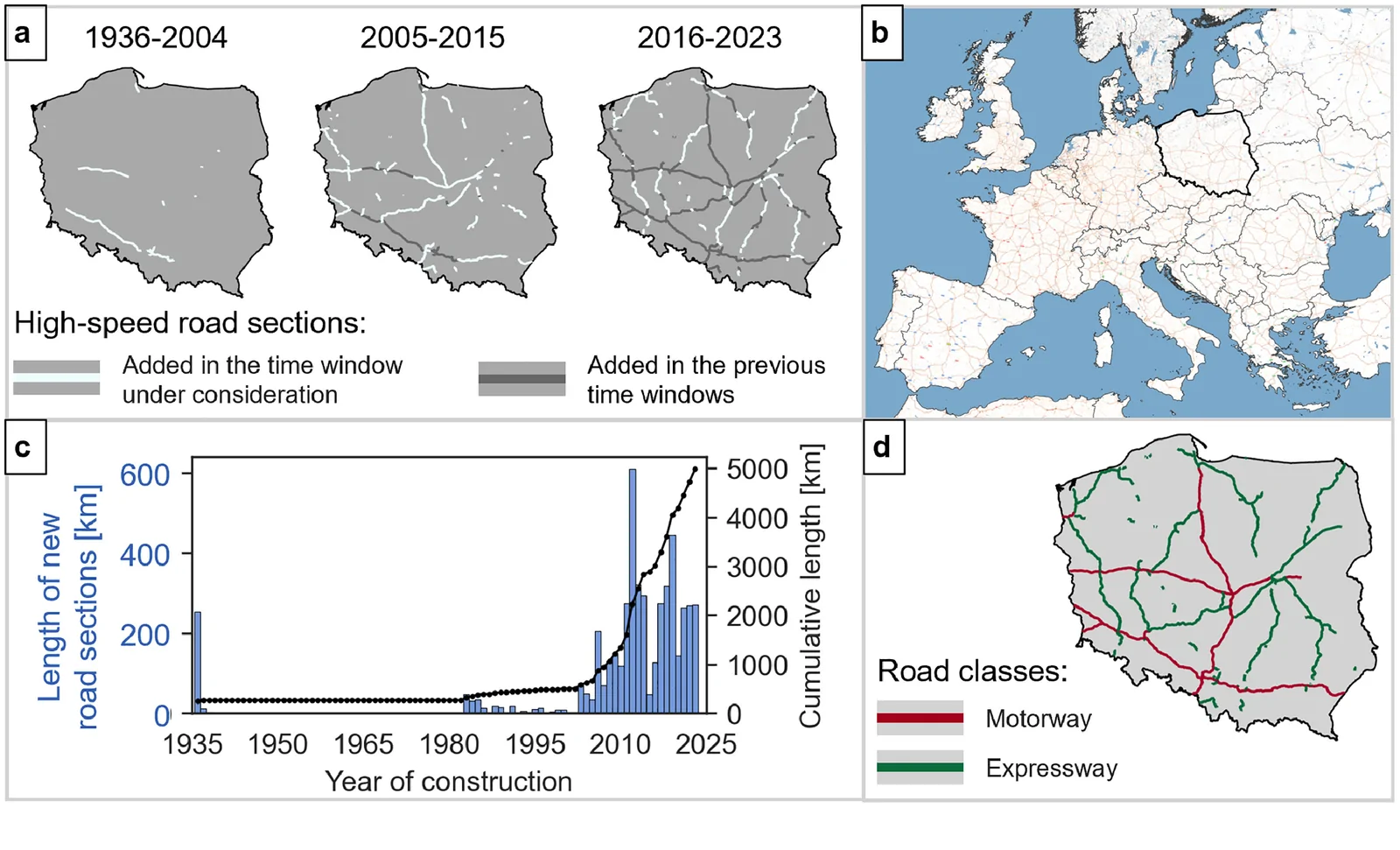
The HiRoN-PL v1.1 high-speed road network dataset: (a) Temporal evolution of the high-speed road network in Poland between 1936 and 2023. (b) Location of Poland within Europe. (c) Annual length (km) of high-speed road network sections commissioned between 1936 and 2023. (d) Public road classes included in the HiRoN-PL v1.1 high-speed road network. Source: Country borders from GADM 4.1^2^, background roads for 2018 from ESRI Global Road Network^3^, and land mask from GHS-LAND R2022A^4^.

The main component of the HiRoN-PL v1.1 dataset is a GeoPackage file with a vector layer of attributed geometries of around 5,000 km of high-speed roads in Poland (Fig. 1). This vector layer comprises of 33,529 line features, projected in the Polish national coordinate reference system (ETRS89 / Poland CS92, EPSG:2180).

**Fig. 2:**
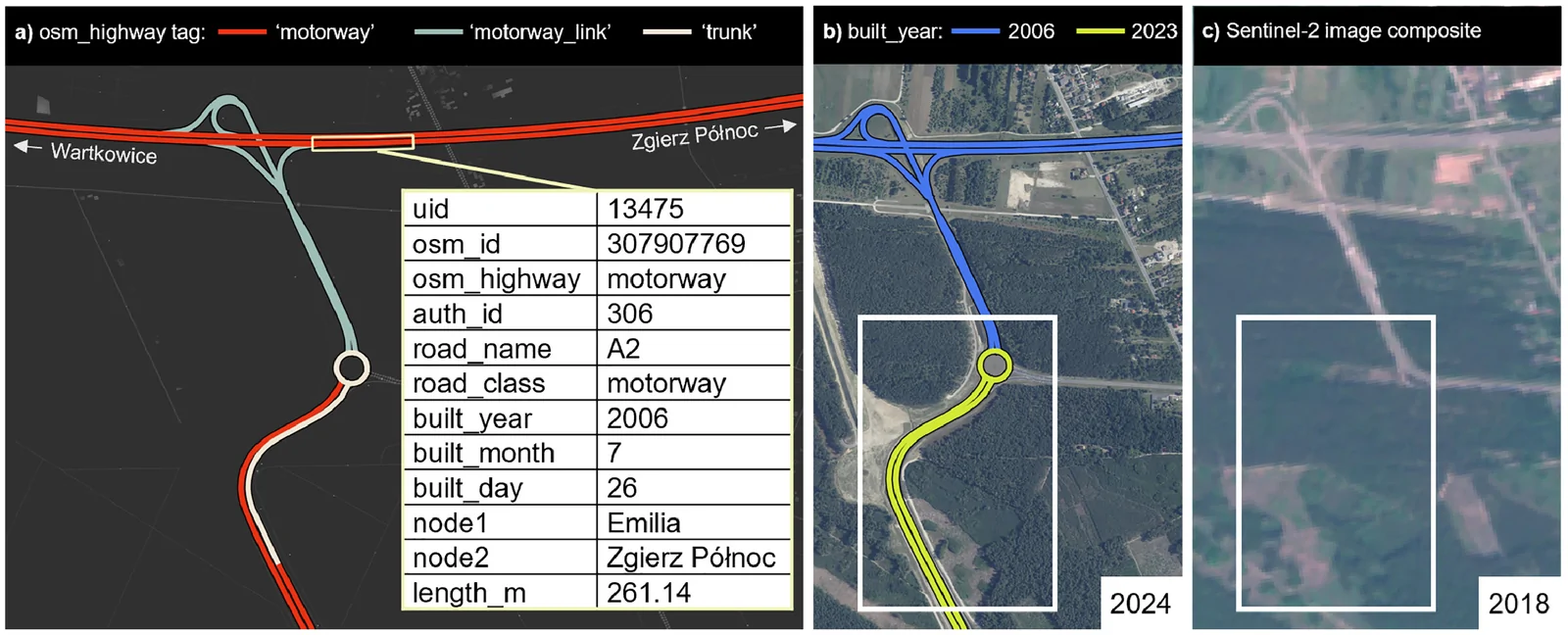
View of the ‘Emilia’ road junction: (a) Attributes for the A2 motorway section, marked in yellow. The colour of the road network indicates the source way object from the OpenStreetMap (OSM) geometries. (b) The year of commissioning of individual sections of the road network overlayed on the orthophotomap from 2024. (c) Sentinel-2 image composite for year 2018. The white box marks the area with the high-speed road network built in 2023, and therefore absent in the 2018 satellite image composite. Source: orthophotomap from Head Office of Geodesy and Cartography (GUGiK)^6^, Sentinel-2 image composite from GHS-composite-S2 R2020A^7^.

The HiRoN-PL v1.1 road network data are based on the vector geometries of OSM ‘*way’* objects representing high-speed roads (Fig. 2). Each HiRoN-PL road network section is attributed with the date of commissioning (year, month, day), the road name, the names of the road junctions^5^ and the road class, consistent with the classification of high-speed roads in Poland (‘*motorway’* or ‘*expressway’,* Fig. 1d). OSM road sections were selected based on their agreement with the authoritative data obtained from GDDKiA, representing the spatial network of motorways and expressways in Poland. The identifiers of the input data and OSM highway tags are preserved to ensure traceability. Each HiRoN-PL road section has a unique internal identifier, and its length in metres is calculated in the national Polish coordinate reference system “Poland CS92” as a derived attribute. An overview of the attributes in HiRoN-PL is provided in Table 1.

**Table 1:** Description of the attributes included in the spatial layer representing road network in the HiRoN-PL v1.1 dataset (DOI: 10.5281/zenodo.19570041).

Field name	Field type	Description
uid	Integer	Unique identifier of a road section.
osm_id	String	Unique way object identifier in OSM. All way objects in OSM have a unique ID describing the type and version of object that constitutes a stable direct link to the OSM database: https://www.openstreetmap.org/way/<osm_id> (example link to way object with osm_id = 4443431).
osm_highway	String	Highway tag of OSM way object (*‘motorway’, ‘motorway_link’* or *‘trunk’*).
auth_id	Decimal	Unique identifier of a road section that constitutes a link to the road geometry in the authoritative data.
road_name	String	Road name of the corresponding road section in the authoritative data.
road_class	String	Road class (‘*motorway’* or ‘*expressway’*) of the corresponding road section in the authoritative high-speed road network data.
built_year	Integer	Year of commissioning (YYYY).
built_month	Integer	Month of commissioning (MM). A value of ‘0’ indicates that the commissioning month was not specified.
built_day	Integer	Day of commissioning (DD). A value of ‘0’ indicates that the commissioning day was not specified.
node1	String	The name of the road junction marking the starting node of the reference road segment.
node2	String	The name of the road junction marking the ending node of the reference road segment.
length_m	Decimal	Length of the road section in metres.

## 4 Experimental Design, Materials and Methods

**Fig. 3:**
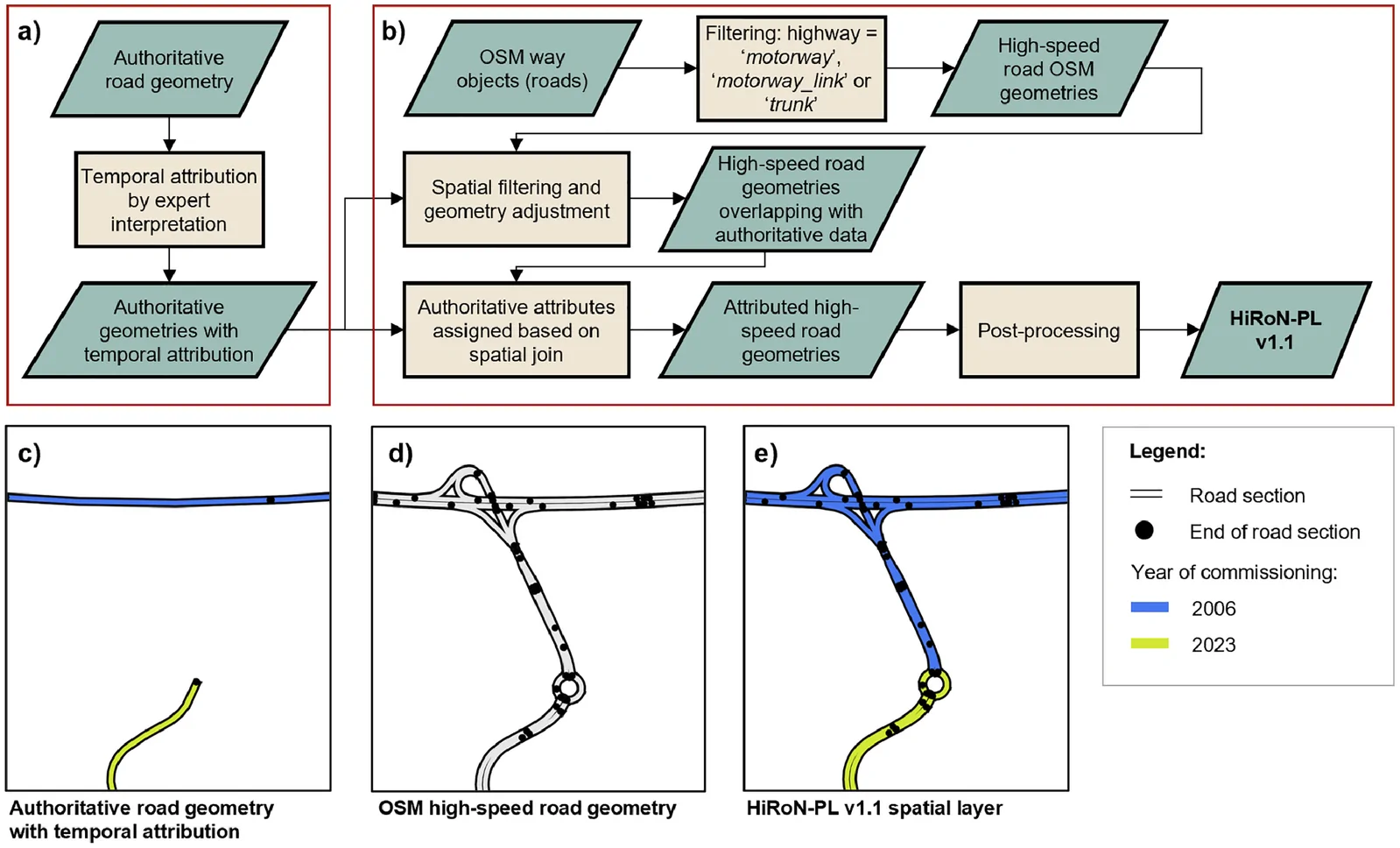
Processing workflow for the production of the HiRoN-PL v1.1 dataset. (a) Workflow for temporal attribution of road geometries based on expert interpretation. (b) Workflow for assigning authoritative and temporal attributes to road geometries derived from OpenStreetMap (OSM). Panels (c)–(e) showcase input and output data on the example of the “Emilia” motorway junction: (c) Authoritative road geometries attributed with the date of commissioning and coloured by year, (d) OSM road network for the same node, (e) HiRoN-PL v1.1 spatial layer for the same node, with the year of commissioning shown in colour. The legend applies to panels (c)–(e).

The production of the HiRoN-PL v1.1 dataset (Table 5) consisted of two main steps: (i) temporal attribution of the high-speed road network by assigning commissioning dates to authoritative road geometries (Fig. 3a), and (ii) integration of these authoritative attributes with geometrically detailed road data derived from OSM (Fig. 3b).

(i) Assigning a commissioning date to authoritative road geometries

The authoritative road geometry data include a spatial layer depicting the state of the motorway and expressway network in Poland in 2023, which partially contained construction dates for the period 2019–2022. These data have been topologically verified and then supplemented with commissioning dates for unattributed or earlier road sections. Commissioning dates have been assigned based on the comparison and expert interpretation of multiple sources, including official information from the GDDKiA, publicly available datasets and regional information portals. Where possible, the assigned date corresponds to the opening of both carriageways without major traffic restrictions. Major road renovations were not taken into account during assignment (e.g. on roads built before the Second World War), and the earliest date on which the road was commissioned was retained.

(ii) Processing of OSM-derived road geometries and integration with authoritative data

The authoritative road geometries are represented by simplified centreline segments (Fig. 3c), which limits their spatial detail. Therefore, to obtain a geometrically refined representation of the high-speed road network, OSM data were used as the primary source of detailed road geometries.

The OSM-derived road network was extracted from ‘*way’* objects corresponding to high-speed roads in Poland, specifically those tagged as ‘*motorway’*, ‘*motorway_link’* and ‘*trunk’*. These objects generally correspond to the Polish national classification of motorways and expressways. To limit the inclusion of irrelevant OSM objects, only ‘*trunk’* road sections located within a 40-m buffer zone around the authoritative road geometries were retained. A 40-m buffer was used to account for dual carriageways, which are represented as separate lines in OSM but as a single centreline in the authoritative data. The selected ‘*trunk’* road sections were combined with all ‘*motorway’* and ‘*motorway_link’* objects to form the input OSM-based road network geometry.

To align the detailed OSM-based geometries with the commissioning phases defined by the authoritative dataset, a spatial matching and segmentation procedure was applied. First, candidate OSM sections were identified for each authoritative road segment using a maximum distance threshold of 1,000 m. A maximum distance of 1,000 m was employed to accommodate curved junction geometries (*‘motorway_link’* objects) extending from their corresponding road segments, whilst precluding the inclusion of unattributed ‘trunk’ and ‘motorway’ sections in the dataset. Initial visual inspection indicated that the 1,000 m threshold value represented a conservative balance between these requirements. The sensitivity analysis supported this choice (Table 2). In the tested range (250–2,000 m), threshold values lower than 750 m overly constrained the representation of ‘*motorway_link’* geometries, whereas larger thresholds progressively extended motorway geometries beyond the spatial extent of the corresponding authoritative sections.

**Table 2:** Sensitivity analysis for maximum distance threshold values compared to the default value (1,000 m; in bold) used in the HiRoN-PL v1.1 production.

Maximum distance threshold value [m]	Difference in the total length as compared to HiRoN-PL v1.1 [%]	Difference in the motorway link length as compared to HiRoN-PL v1.1 [%]
250	-1.4	-6.9
500	-0.5	-1.5
750	-0.2	-0.3
**1,000**	**0.0**	**-**
1,250	0.2	0.2
1,500	0.3	0.3
1,750	0.4	0.3
2,000	0.6	0.5

To ensure geometric consistency, an additional angular filter was applied, restricting matches to sections with an azimuth difference of less than 30°. Sensitivity analysis showed that a more restrictive choice of threshold value resulted in the omission of authoritative road segments, and that the chosen threshold value had a marginal impact on the total length of the road network (Table 3).

**Table 3:** Sensitivity analysis for azimuth difference threshold values compared to the default value (30°; in bold) used in the HiRoN-PL v1.1 production.

Azimuth difference threshold value [°]	Number of unmatched authoritative road segments	Difference in the total length as compared to HiRoN-PL v1.1 [%]
10	5	0.004
20	2	0.001
**30**	**1**	***-***
40	1	0.007
50	1	0.022

OSM candidate sections meeting these two criteria were used for segmentation. The endpoints of matched authoritative segments were snapped onto the corresponding OSM geometries, and the OSM lines were split at these snapped locations. This procedure resulted in a set of road sections generated from OSM data (Fig. 3d), which spatially correspond to the segments of high-speed roads from the authoritative data (Table 4).

**Table 4:** Comparison of lengths of authoritative high-speed road segments and the OSM-derived HiRoN-PL v1.1 road network. For both datasets, lengths are reported without motorway links.

Total length of HiRoN-PL v1.1 without ‘motorway_link’ objects	Total length of authoritative road segments	Number of unmatched authoritative road segments	Length of unmatched authoritative road segments
4,992.8 km	4,941.9 km	1	0.1 km

Each resulting OSM-based section was assigned attributes from the nearest authoritative road section using a nearest-neighbour spatial join based on section midpoints. This enabled the transfer of authoritative attributes, namely: road identifiers, classes, segment names, names of the road junctions that designate the starting and ending nodes of each segment, as well as temporal attributes, including the year, month, and day of commissioning (Fig. 3e). Sections to which no year of commissioning was assigned were excluded from further processing. The resulting high-speed road network includes links to geometries sourced from OSM via the ‘*osm_id’* and ‘*osm_highway’* attributes, as well as links to authoritative road sections via the ‘*auth_id’* attribute. Section length (‘*length*_*m*’) was calculated from the final geometry.

To ensure attribute consistency, ‘*motorway_link*’ objects forming motorway junctions were aggregated and jointly processed based on the assumption that a high-speed road junction constitutes part of this road. Objects within a single junction were grouped using a graph-based method, and their temporal attributes and those derived from authoritative data were harmonised to the section with the earliest opening date within each group (see, for example, Fig. 2c: the 2006 motorway link connects the expressway built in 2023).

The resulting HiRoN-PL v1.1 dataset provides a detailed spatial representation of the evolution of the high-speed road network in Poland (Fig. 3e), combining detailed OSM geometry, authoritative attributes, and temporal information regarding the commissioning of individual road sections. The workflow summary of HiRoN-PL v1.1 production is provided in Table 5 and the processing scripts are available in the GitHub repository: https://github.com/katarzynagoch/hironpl. The current release (v1.1) is archived and versioned in Zenodo (10.5281/zenodo.19570041), with a persistent concept DOI providing access to all versions (10.5281/zenodo.16536448). Future updates are anticipated every 2–3 years to incorporate newly commissioned roads and methodological improvements, with long-term maintenance supported by the Institute of Geography and Spatial Organization, Polish Academy of Sciences.

**Table 5:** Summary of the processing workflow used to produce the HiRoN-PL v1.1 dataset, including input data, processing steps, key parameters and criteria, and resulting outputs.

Step	Input	Processing	Key parameters/criteria	Output
**1**	Authoritative road network	Topological verification	Correct topology at road junctions	Topologically verified authoritative network
**2**	Historical sources: GDDKiA information, public datasets and regional information portals	Expert interpretation of commissioning phases	Earliest opening date of both carriageways; road renovations excluded	Temporally attributed authoritative road segments
**3**	OSM road network	Selection of high-speed road sections	*‘motorway’, ‘motorway_link’* and *‘trunk’* within 40 m of authoritative centrelines	Selected OSM high-speed road sections
**4**	Authoritative and OSM road networks	Spatial matching	Distance ≤1,000 m; azimuth difference <30°	Candidate OSM road sections
**5**	Authoritative road segments and candidate OSM sections	Snapping and segmentation	Authoritative segment endpoints snapped to OSM geometries	OSM sections segmented according to authoritative road segments
**6**	Segmented OSM network and authoritative road segments	Attribute transfer	Nearest segment based on midpoint distance; maximum distance 1,000 m	OSM sections with authoritative and temporal attributes
**7**	Attributed OSM road sections	Junction harmonisation	Earliest commissioning date within each junction group	Harmonised OSM road network
**8**	Harmonised road network	Quality control and validation	Comparison with reference data and other datasets	HiRoN-PL v1.1

**Fig. 4:**
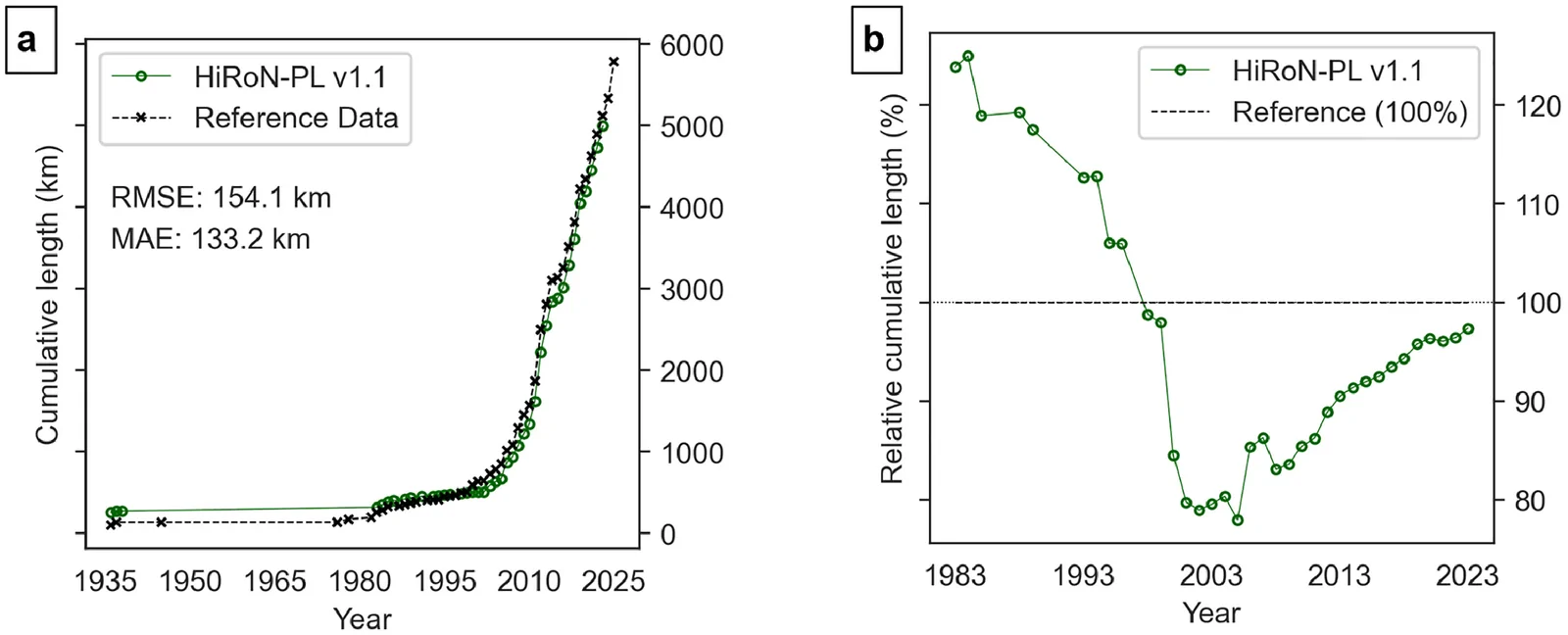
(a) Cumulative length of high-speed roads in the HiRoN-PL v1.1 dataset, shown as absolute values, compared with reference nation-level statistics on the high-speed road network. Error values ​​are given for the reference observation years. (b) Cumulative length of high-speed roads in the HiRoN-PL v1.1 dataset, shown as relative values for the period 1983–2023.

To assess the accuracy of the assigned commissioning dates, the lengths of motorways and expressways in the HiRoN-PL v1.1 dataset were compared with national-level reference summaries of the high-speed road network in Poland^8^. Both sources show a similar temporal pattern, with limited growth until 1983, followed by gradual expansion over the next two decades and marked acceleration after 2004 (Fig. 4a). Across the reference observation years, the mean absolute error (MAE) and root mean square error (RMSE) were 133.2 km and 154.1 km, respectively. Differences in cumulative road length varied over time, from HiRoN-PL exceeding the reference values by approximately 20% in the earlier years to underestimating them by approximately 20% during the 2000s. This discrepancy subsequently decreased over time (Fig. 4b).

**Fig. 5:**
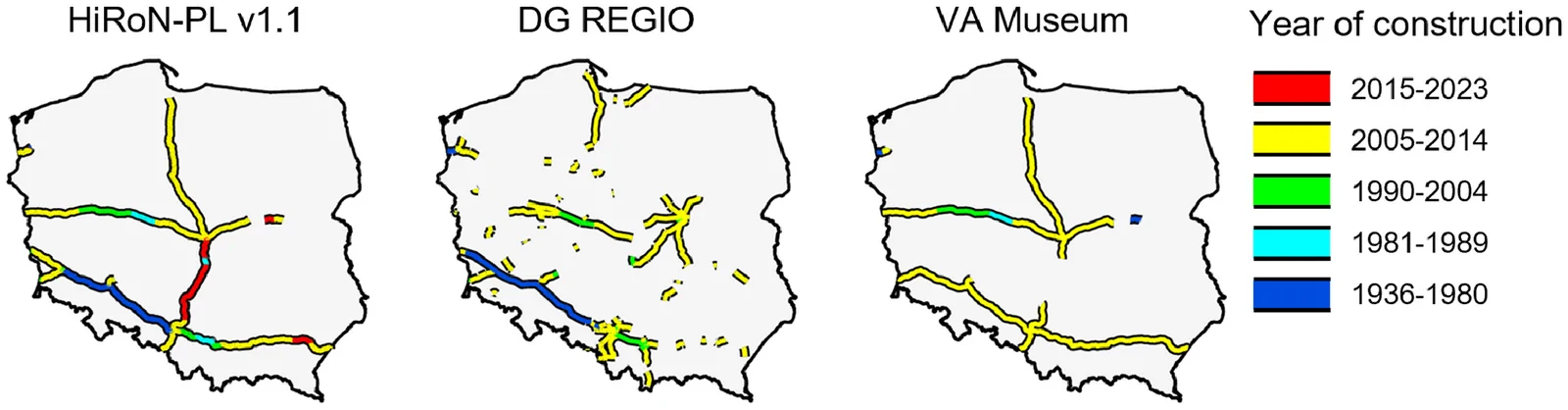
Temporal evolution of the motorway network based on roads selected from three datasets: roads classified as ‘motorways’ in the HiRoN-PL v1.1 dataset; roads classified as ‘highways’ in the historical road network dataset developed by the Directorate-General for Regional and Urban Policy of the European Commission (DG REGIO); and all roads from the VA Museum dataset, commissioned by the Victoria and Albert Museum, London.

To assess the added value of the HiRoN-PL v1.1 dataset, we compared it with two pan-European datasets measuring the evolution of motorway networks, available under open-access licences (Fig. 5). The dataset compiled by the European Commission’s Directorate-General for Regional and Urban Policy (DG REGIO) is derived from digitised historical maps and road atlases [16]. The dataset compiled by the Victoria and Albert Museum in London (VA Museum) is the result of combining crowdsourced data (OSM) and authoritative road geometries into a motorway road network dataset [17]. In the VA Museum dataset, the construction epoch of motorway segments was estimated based on expert interpretation. Both the DG REGIO dataset and the VA Museum dataset use different classifications of the road network: in the DG REGIO product, the road network is classified into three broad categories: ‘*highways’*, ‘*main roads’* and ‘*secondary roads’*; whereas the VA Museum dataset covers only the ‘*motorway’* road class. The HiRoN-PL dataset follows a national classification of the public road network in Poland, which includes both motorways and expressways within the high-speed road category (Fig. 1d).

These semantic differences in the definition of high-speed roads are evident from the spatial comparison across Poland (Fig. 5). Roads classified as ‘*motorways’* in the HiRoN-PL v1.1 show a spatial pattern similar to that of the VA Museum dataset, as both datasets are based on OSM geometries. In contrast, roads classified as ‘*highways’* in the DG REGIO dataset show a more scattered spatial pattern, reflecting differences in road classification related to the manual digitisation of road geometries used in its production.

For Poland, HiRoN-PL v1.1 offers the longest time series and the highest temporal resolution among the compared datasets, and deviates least from the total reference length of the high-speed network (Table 6). These properties, coupled with the distinction between ‘*motorways’* and ‘*expressways’* in the ‘*road_class’* attribute, make the HiRoN-PL dataset highly useful for national-level applications compared to other existing pan-European products.

**Table 6:** Cross-comparison of three datasets representing the evolution of motorway networks: HiRoN-PL v1.1. (this study); the historical road network dataset developed by the Directorate-General for Regional and Urban Policy of the European Commission (DG REGIO); and the VA Museum dataset, commissioned by the Victoria and Albert Museum, London. The DG REGIO and VA Museum datasets were clipped to the territory of Poland prior to the analysis. Road length at the latest common date was computed for all roads in the HiRoN-PL v1.1 and the VA Museum dataset, and for roads classified as ‘highways’ in the DG REGIO dataset.

Dataset	HiRoN-PL v1.1	DG REGIO	VA Museum
Earliest year represented	1936	1955	1978
Latest year represented	2023	2012	2014
Temporal span	88 years	58 years	37 years
Number of distinct years/epochs	40	5	10
Road length at latest common date (2012)	2,219.9 km	3,929.9 km	1,926.6 km
Deviation from reference high-speed road network length in 2012 (2,495 km)	-275.1 km	1,434.9 km	-568.4 km
Relative deviation from reference high-speed road network length in 2012 (2,495 km)	-11 %	57.5 %	-22.8 %

## Limitations

Due to the nature of the data, the HiRoN-PL v1.1 dataset is affected by uncertainties regarding the attribution of commissioning dates for individual sections of expressways and motorways. In particular, in cases of reconstruction, modernisation or phased expansion, the earliest identifiable opening date for the section in question has been assigned. Consequently, the reported commissioning dates should be interpreted as an approximation rather than an exact record. Discrepancies may relate to the date when the main section was opened to traffic, which does not always coincide with the date when the road became fully operational. In cases of discrepancy, the earlier date was used whenever it could be reliably identified. The HiRoN-PL dataset is primarily intended to support long-term transport analyses spanning decades, for which uncertainties of a few days or weeks are unlikely to substantially affect the results. Nevertheless, these temporal uncertainties should be considered in applications requiring finer temporal precision, such as local travel-time analyses and traffic-speed modelling. In future releases of HiRoN-PL, we intend to include an estimate of temporal uncertainty, for example, by considering the time range of commissioning dates provided by different sources.

Moreover, certain limitations arise from the procedure used to attribute commissioning dates to road junctions. In HiRoN-PL v1.1, road sections constituting a junction were grouped and assigned the earliest opening date among the sections within each group. This approach enables the integration of complex OSM geometries with simplified authoritative data. However, it introduces some uncertainty, as the assumption that all components of a junction share the earliest opening date may not always hold. In particular, junctions constructed after the corresponding road segment may be (incorrectly) assigned an earlier commissioning date, potentially affecting routing analyses based on historical network conditions. This limitation will be addressed in future releases of the dataset through more detailed verification of junction commissioning dates.

Additional uncertainties arise from the use of OSM data as a source of detailed road geometry. Although OSM provides high spatial detail and extensive coverage, it is a crowdsourced dataset and may contain inaccuracies in geometry, classification or data completeness, as well as topological inconsistencies transferred to the resulting dataset. In the case of Poland, OSM road features are estimated to be highly complete [18]. Although at the local level the completeness of OSM features varies and correlates with the economic development of individual municipalities, it is estimated that the coverage of OSM road objects in terms of total length is over 70% [19].

## Ethics Statement

The current work does not involve human subjects, animal experiments, or any data collected from social media platforms.

## CRediT Author Statement

**Katarzyna Krasnodębska**: Conceptualisation, Methodology, Software, Writing - Original Draft, Writing - Review & Editing. **Przemysław Śleszyński:** Data curation, Funding acquisition, Investigation, Writing - Review & Editing, Resources. **Johannes H. Uhl:** Writing - Review & Editing. **Łukasz Kubiak:** Resources.

## Declaration of Generative AI and AI-assisted Technologies in the Manuscript Preparation Process

During the preparation of this work, the author(s) used editGPT for language correction. The author(s) reviewed and edited the output as needed and take full responsibility for the content of the published article.

## Data Availability

ZenodoHiRoN-PL: High-Speed Road Network Vector Data with Construction Year Attribution for Poland (1936-2023) (Original data).

## Footnotes

1 The term “high-speed” refers to national roads that belong to the highest class of public roads in Poland: motorways and expressways. Both classes of roads have dual carriageways and are intended exclusively for motor vehicle traffic, with speed limits typically of 100 km/h or higher.

## Footnotes

2 https://gadm.org/index.html.

3 https://services.arcgisonline.com/ArcGIS/rest/services/Reference/World_Transportation/MapServer.

4 https://human-settlement.emergency.copernicus.eu/ghs_land2022.php.

5 Road junctions are named after nearby cities or towns in accordance with the order of the General Director of National Roads and Motorways of 7 April 2009 on the establishment of directional signage for national roads.

6 https://www.geoportal.gov.pl/en/data/orthophotomap-orto/.

7 https://human-settlement.emergency.copernicus.eu/ghs_s2composite.php.

8 The reference figures for the total length of the high-speed road network in Poland were obtained from Wikipedia (https://pl.wikipedia.org/wiki/Autostrady_i_drogi_ekspresowe_w_Polsce). These figures are based on estimates included in the National Road Construction Programme for 2014–2023 (Resolution No. 108/2019 of the Council of Ministers of 24 September 2019), the annual reports of GDDKiA and media reports.
